# Experience Sampling-Based Personalized Feedback and Positive Affect: A Randomized Controlled Trial in Depressed Patients

**DOI:** 10.1371/journal.pone.0128095

**Published:** 2015-06-02

**Authors:** Jessica A. Hartmann, Marieke Wichers, Claudia Menne-Lothmann, Ingrid Kramer, Wolfgang Viechtbauer, Frenk Peeters, Koen R. J. Schruers, Alex L. van Bemmel, Inez Myin-Germeys, Philippe Delespaul, Jim van Os, Claudia J. P. Simons

**Affiliations:** 1 Department of Psychiatry and Psychology, Maastricht University Medical Centre, European Graduate School of Neuroscience, SEARCH, Maastricht, The Netherlands; 2 GGzE, Institute of Mental Health Care Eindhoven and the Kempen, Eindhoven, The Netherlands; 3 Orygen, the National Centre of Excellence in Youth Mental Health, University of Melbourne, Melbourne, Australia; 4 Center for Learning and Experimental Psychology, Faculty of Psychology, Leuven University, Leuven, Belgium; 5 Mondriaan Mental Health Trust, South Limburg, The Netherlands; 6 King’s College London, Department of Psychosis Studies, Institute of Psychiatry, London, United Kingdom; Erasmus University Rotterdam, NETHERLANDS

## Abstract

**Objectives:**

Positive affect (PA) plays a crucial role in the development, course, and recovery of depression. Recently, we showed that a therapeutic application of the experience sampling method (ESM), consisting of feedback focusing on PA in daily life, was associated with a decrease in depressive symptoms. The present study investigated whether the experience of PA increased during the course of this intervention.

**Design:**

Multicentre parallel randomized controlled trial. An electronic random sequence generator was used to allocate treatments.

**Settings:**

University, two local mental health care institutions, one local hospital.

**Participants:**

102 pharmacologically treated outpatients with a DSM-IV diagnosis of major depressive disorder, randomized over three treatment arms.

**Intervention:**

Six weeks of ESM self-monitoring combined with weekly PA-focused feedback sessions (experimental group); six weeks of ESM self-monitoring combined with six weekly sessions without feedback (pseudo-experimental group); or treatment as usual (control group).

**Main outcome:**

The interaction between treatment allocation and time in predicting positive and negative affect (NA) was investigated in multilevel regression models.

**Results:**

102 patients were randomized (mean age 48.0, SD 10.2) of which 81 finished the entire study protocol. All 102 patients were included in the analyses. The experimental group did not show a significant larger increase in momentary PA during or shortly after the intervention compared to the pseudo-experimental or control groups (χ^2^ (2) =0.33, *p*=.846). The pseudo-experimental group showed a larger decrease in NA compared to the control group (χ^2^ (1) =6.29, *p*=.012).

**Conclusion:**

PA-focused feedback did not significantly impact daily life PA during or shortly after the intervention. As the previously reported reduction in depressive symptoms associated with the feedback unveiled itself only after weeks, it is conceivable that the effects on daily life PA also evolve slowly and therefore were not captured by the experience sampling procedure immediately after treatment.

**Trial Registration:**

Trialregister.nl/trialreg/index.asp. NTR1974

## Introduction

Research on the factor structure of human emotional responses has identified two different, yet related, affective dimensions: Positive (PA) and negative affect (NA). The reward-oriented PA dimension, reflecting affective states such as enthusiasm, cheerfulness, and contentment, is associated with behavioural approach or engagement with the environment; the threat-oriented NA dimension, reflecting affective states such as anxiety, loneliness, and guilt, is associated with behavioural avoidance or withdrawal [1, 2]. PA and NA seem to represent two separate dimensions, which are usually negatively correlated within a person [3].

Traditionally, depression has been linked to disturbances in NA regulation. Stress-sensitivity, as an example, has been intensively investigated as a prominent (risk-) factor in depression [4]. Similarly, cognitive behavioural therapy traditionally focusses on negative thoughts and the down-regulation of NA [5]. However, converging evidence suggests that particularly a deficiency in PA, linked to the concept of anhedonia, represents a core mechanism underlying depression [6]. Likewise, recent studies show that a high ability to experience PA, rather than NA, is predictive for the development, course, and recovery of depression [7–11]. This finding provides potential routes to novel treatments in depression, i.e., interventions aimed at increasing positive affective experience in daily life [12].

### Experience Sampling Methodology as Intervention

The Experience Sampling Method (ESM) is a structured diary technique requiring individuals to repeatedly report their affective states and behaviour together with contextual information in the flow of daily life [13–15]. ESM has until recently been mainly used to *assess* subjects for research purposes. However, the replacement of paper-and-pencil based diaries by electronic sampling devices introduces the possibility of rapid analysis and availability of real life experiential data [16]. Electronically collected data thus can be used not only to monitor, but also to give feedback to the patient and professional caregiver with minimal delay, providing fine-grained, prospective information on affective patterns, behaviour and contextual influences in daily life [17, 18]. Using ESM not merely as an instrument to monitor, but as a tool to *intervene* (referred to as ESM-I), may be promising for the development of new mHealth (‘mobile health’) therapies in depression [17–19].

### The present study

The current randomized controlled trial (RCT) in a sample of depressed patients was designed to evaluate an intervention aimed at reducing depressive symptoms by enhancing PA in daily life using ESM-I. During the six-week intervention, self-collected ESM data on momentary affective and behavioural responses was fed-back to the patient on a weekly basis. Given the potentially pivotal role of PA in the recovery of depression, the feedback focused on positive affective experience in daily life. The experimental feedback condition was compared to two other conditions: a pseudo-experimental condition in which the weekly feedback sessions were replaced by weekly meetings with the researcher without feedback, and a control condition without any meetings with the researcher during the intervention period.

Recently, we reported the results of this RCT with respect to its effectiveness in reducing depressive symptomatology [20]. It was demonstrated that the ESM-derived, PA-focused feedback was associated with a linear decrease in depressive symptoms in the six months following the intervention, a pattern not observed in the other two conditions [20]. As such, the intervention appeared to be effective in reducing depressive symptoms in the long run. However, the question remains as to whether the intervention, aimed at increasing PA in daily life, was indeed associated with a boost of momentary positive affective experience. The purpose of the present study is to shed light on the potential micro-mechanism by which PA-focused ESM-I may lead to a decrease in depressive symptoms. It was hypothesized that the experimental feedback condition, compared to the pseudo-experimental and control condition, would be associated with an increase in PA (and an associated decrease in NA) during and shortly after the intervention.

## Methods

### Ethics statement

The study was approved by the local ethics committee (Medical Ethics Committee of Maastricht University Medical Centre, azM/UM, #NL26181.068.09/MEC09-03-013) and all participants provided written informed consent before their enrolment. The trial was registered at the Netherlands Trial Register (Identifier: NTR1974). The study was performed according to the declaration of Helsinki. The original protocol and a CONSORT checklist are provided (see S1 Protocol and S1 CONSORT Checklist).

### Study Overview

The multicentre trial was conducted between January 2010 and October 2012 in the following settings: Maastricht University, two local mental health care institutions, and a local hospital. Participant recruitment took place between January 2010 and February 2012 in mental health care institutions and through local advertisements.

Participants were considered eligible when they were between 18 and 65 years of age; diagnosed with a depressive episode according to DSM-IV [21] with current or residual symptoms (17-item Hamilton Depression Rating Scale (HDRS) score of >7); treated with antidepressants or mood stabilizers; and in possession of sufficient Dutch language skills. Participants were excluded if they met criteria for a non-affective psychotic disorder according to DSM-IV or if they reported a (hypo-) manic or mixed episode within the past month.

### Procedure

The recruitment process started with a short telephone interview conducted by a psychologist or psychiatrist to establish whether inclusion criteria were likely met. If so, potential participants were invited to a full screening on site. During the screening, the Structured Clinical Interview for DSM-IV Axis I Disorders (SCID-I) [21] and the HDRS [22] were administered. If admitted to the study, the participants underwent a five-day ESM baseline assessment followed by randomization to either the experimental group, pseudo-experimental group or control group. Randomization with allocation ratio 1:1:1 was stratified with respect to duration of pharmacological treatment (use of a particular antidepressant for shorter vs. longer than 8 weeks prior to study entry) and the attendance of psychotherapy (yes/no). Allocation took place by drawing a sealed envelope (prepared by an independent research coordinator) with a number sequence produced by an electronic random sequence generator (www.random.org) in blocks of six. Due to the nature of the psychological treatment allocation was not blinded.

Participants allocated to the experimental group underwent a six-week ESM protocol consisting of a three-day ESM assessment period per week and, in addition to their treatment as usual (TAU), six weekly ESM-based feedback sessions (ESM-I). Participants allocated to the pseudo-experimental group followed the same protocol, but instead of receiving ESM-based feedback, this group engaged in a structured conversation with the researcher in the form of an HDRS interview during the weekly sessions. The rationale for including the pseudo-experimental group was to control for the potential effects of prolonged ESM-monitoring and weekly appointments with the researcher on mood. During the six-week ESM protocol of the experimental and pseudo-experimental groups, participants allocated to the control group received TAU only. Finally, all participants underwent a five-day ESM period after the six-week intervention period (hereafter: post-assessment).

In summary, all three groups underwent an ESM pre- and post-assessment. Based on previous work and conventions regarding experiencing sampling research in individuals with mental illness [23, 24], the duration of the pre-and post-assessment was set to five consecutive days. During the six-week intervention period, the experimental and pseudo-experimental groups engaged in three days of ESM per week (i.e., three consecutive days instead of five days a week in order to reduce the weekly burden imposed on the patients), combined with weekly feedback sessions (experimental group) or weekly structured conversations without feedback (pseudo-experimental group).

### Experience Sampling Method

ESM is a validated, structured diary technique to assess participants in their daily living environment. It is a momentary assessment method providing repeated in-the-moment micro-measurements of affect and context in a prospective and ecologically valid manner [13, 14, 23].

Participants received a dedicated mHealth electronic ESM device (‘PsyMate’), emitting a signal (‘beep’) at a random moment in each of ten 90-minute time blocks between 07:30 am and 10:30 pm. The signal prompted participants to fill in self-assessments (7-point Likert scale ratings and forced-choice questions) automatically shown on the device display. To minimize memory distortion, participants were instructed to complete their reports immediately upon the signal; after 10 minutes, the self-assessments were no longer available for completion. The ESM procedure was explained in an initial briefing session and a practice run was performed to ensure that the participants understood the questions and the device.

### Intervention

The experimental group received standardized ESM-derived feedback. Feedback sessions immediately followed the weekly ESM procedure. In these face-to-face sessions, feedback was provided by a psychologist or psychiatrist. The feedback on participants’ momentary affective states in specific daily life contexts and the association with depressive symptoms was given graphically, presented by the researcher according to a standardized verbal protocol. A written summary based on a standardized template was provided to the participant as well as the attending clinician.

#### Feedback structure

Every feedback session consisted of two parts. In the first part, information with respect to experiences during the most recent week was presented. In the second part, this information was placed in the context of all previous weeks.

#### Feedback content

The feedback sessions consisted of information that was repeated each week, as well as variable feedback elements. Each week, information regarding the degree and distribution of PA experienced during the past week was presented, as well as graphs displaying the course of experienced PA across the weeks. Furthermore, the course of depressive feelings over the weeks was presented in each session and compared to the course of PA. The variable elements in the feedback procedure were divided over three modules. The first two weeks focused on daily activities and PA experienced during particular activities. The third and fourth week focused on daily events and PA associated with these events. The last two weeks focused on social interaction and associated PA. Variable elements were presented as described above for the fixed feedback information, focusing on the most recent week followed by longitudinal information over the weeks.

Please see S1 Feedback for an example of the graphical feedback and how the feedback was discussed with the participant (extract of the session).

### Outcome Measures

Primary outcome measures were PA and NA, derived as follows. During the ESM assessments as described above, participants rated their momentary mental states by rating adjectives (identical at every time point) on a 7-point Likert scale, ranging from 1 (not) to 7 (very). Using the STATA command FACTOR, within-person factor analysis (principal component factors with oblique rotation) performed on these items (centred per person and ESM-week), identified two factors with an eigenvalue >1, accounting for 46% of the variance: positive affect and negative affect (Cronbach’s α 0.74/0.54 respectively). Positive affect comprised the average of ratings on ‘cheerful’, ‘enthusiastic’, ‘relaxed’ and ‘satisfied’ (with factor loadings >.6); negative affect comprised the average of ratings on ‘lonely’, ‘anxious’, ‘guilty’, and ‘suspicious’ (with factor loadings >.5).

### Statistical Analyses

The data set had a hierarchical structure, due to within-person clustering: repeated measurements (level 1), nested within individuals (level 2). Observations within one participant are more similar than those across participants. As nesting of data typically violates the assumption of independent residuals, multilevel regression analysis was used that takes clustering into account [25]. All analyses were performed using STATA 12 [26].

In order to examine the effect of the feedback intervention on PA and NA, two sets of analyses were conducted. In the first set, the effects of the feedback intervention on change in PA and NA from pre- to post-assessment, relative to the two control groups, was examined. To this end, linear multilevel regression analyses (STATA command XTMIXED) were conducted to inspect the categorical by categorical interaction between treatment allocation (experimental vs. pseudo-experimental vs. control group) and time (pre- vs. post-assessment) as fixed effects. The multilevel models for PA and NA, fitted via restricted maximum likelihood, included a random intercept for participant and a random slope for time. The post-estimation commands MARGINS and TEST (providing Wald tests) were used to calculate and compare the effect of time on affect in the different groups.

In the second set of analyses, the effect of the feedback intervention on affect *during* the intervention period, relative to the pseudo-experimental group, was examined. As the control group was not seen during the intervention period, this group is not included in this analysis. Using multilevel regression analyses (STATA command XTMIXED) fitted via restricted maximum likelihood, the categorical by categorical interaction between treatment allocation (experimental vs. pseudo-experimental) and time (in weeks since start of the intervention) as fixed effects was examined; the model included a random intercept for participant and a random slope for time (intervention week 1–6).

Power calculations using the STATA SAMPSI command were based on previous work [27], and led to an initial sample size of 120 with a power of 84% to detect a 3-point difference in the score on the 17-item Hamilton Depression Rating Scale (HDRS, [22]) [28, 29], the main outcome as reported in the previous study [20]. However, because many participants were excluded, the inclusion rate was lower than expected. The eventual number of patients who participated in the trial was 102.

## Results

### Participants

**Fig 1:**
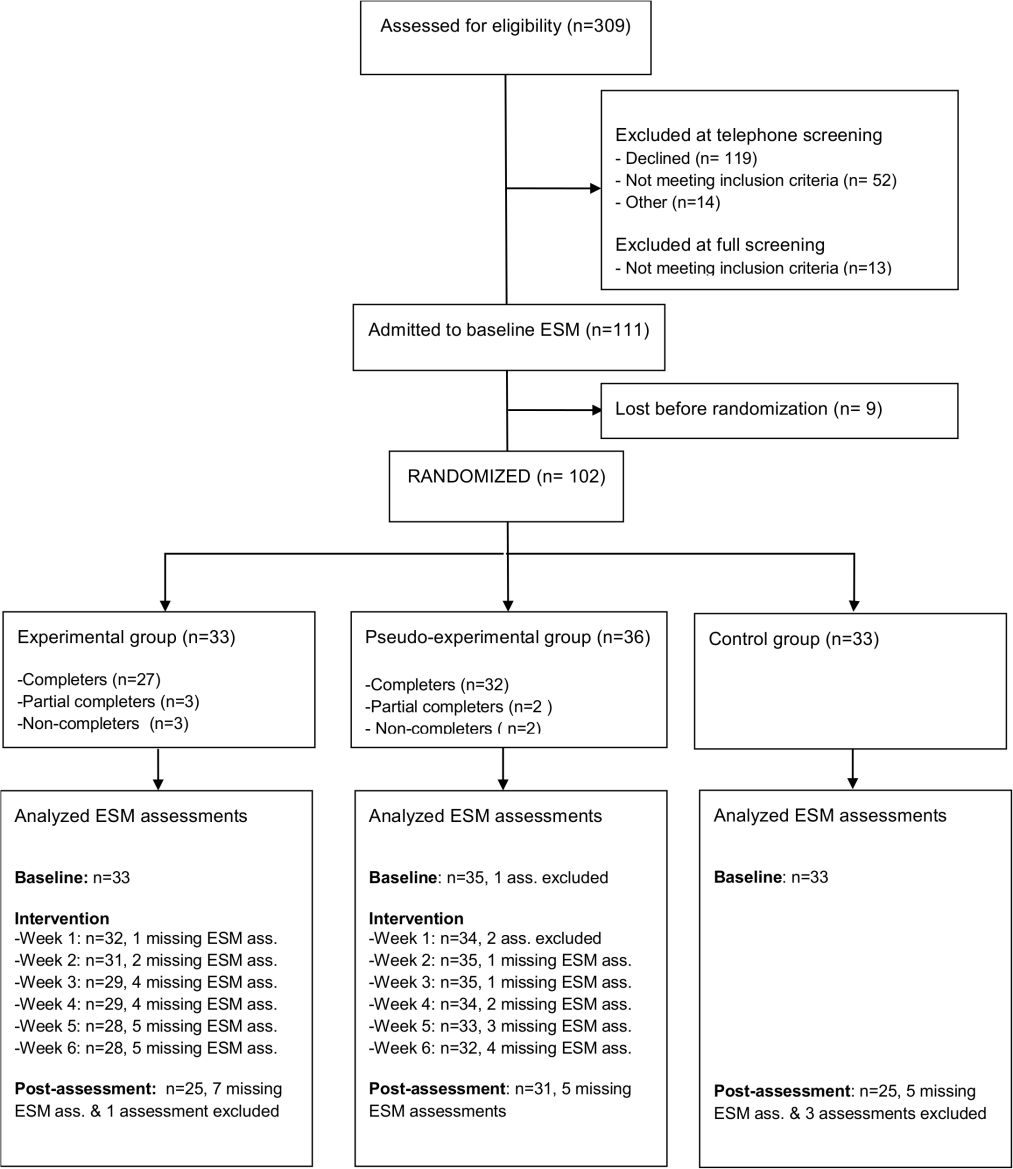
Participant flow diagram. Completer: Participant who attended all six intervention sessions; Partial Completer: Participant who attended less than six intervention sessions but did not withdrew from the study; non-completer: Participant who attended less than six intervention sessions and withdrew from the study.

A total of 309 patients underwent an initial screening by telephone, of which 102 were allocated at random to the experimental, pseudo-experimental or control groups (see Fig 1 for the participant flow through the study). At screening, the groups showed some differences in clinical features: Compared to the pseudo-experimental and control group, patients in the experimental group more often used lithium and displayed lower HDRS scores (Table 1); these differences were no longer significant at baseline (two weeks later, i.e., just before the start of the intervention, see also [20]). There were significant differences in sociodemographic variables between the groups at baseline (Table 1).

**Table 1:** Sample characteristics.

	Experimental Group	Pseudo-experimental Group	Control Group	Total
N	33	36	33	102
Age (SD)	48.7 (10.2)	46.7 (9.6)	48.9 (10.9)	48.0 (10.2)
Sex (M/F)	17/16	14/22	15/18	46/56
Education				
Low (%)	6 (18.2)	9 (25.0)	10 (30.3)	24.5)
Middle (%)	12 (36.4)	14 (38.9)	12 (36.4)	38 (37.3)
High (%)	15 (45.5)	13 (36.1)	11 (33.3)	39 (38.2)
Full/ part time work (%)	13 (39.4)	10 (27.8)	12 (36.4)	35 (34.3)
Married (%)	12 (36.4)	16 (44.4)	17(51.5)	45 (44.1)
PA pre-assessment (SD)	3.4 (1.3)	3.3 (1.3)	3.2 (1.0)	3.3 (1.2)
PA post-assessment	3.7 (1.3)	3.4 (1.4)	3.4 (1.8)	3.5 (1.3)
NA pre-assessment (SD)	2.2 (1.1)	2.2 (1.2)	2.3 (1.2)	2.3 (1.2)
NA post-assessment	2.1 (1.4)	2.1 (1.2)	2.3 (1.3)	2.2 (1.3)
Current psychotherapy	4 (14.8)	4 (13.3)	2 (7.4)	10 (11.76)
Use of antidepressants^a^				
Maintenance (%)	28 (84.9)	30 (83.3)	25 (75.8)	83 (81.4)
Acute phase (%)	5 (15.2)	6 (16.7)	8 (24.2)	19 (18.6)
HDRS Mean (SD)^b^	14.1 (4.5)	16.2 (4.8)	17.0 (4.3)	15.8 (4.6)
Depressive episode^c^				
Current (%)	19 (57.6)	24 (66.7)	25 (75.8)	68 (66.7)
In past (%)	14 (42.4)	12 (33.3)	8 (24.2)	34 (33.3)
Bipolar disorder	5 (15.2)	2 (5.6)	2 (6.1)	9 (8.8)
Use of lithium (%)^d^	7 (21.2)	1 (2.8)	3 (9.1)	11 (10.8)

^a^ Maintenance = use of the antidepressant for ≥ 8 weeks; new = use of antidepressant < 8 weeks

^b^ HDRS (Hamilton Depression Rating Scale) score significantly different between groups at screening (F = 3.64; *p* =. 03)

^c^ According to SCID (Structured Clinical Interview for DSM-IV-TR Axis I disorders)

^d^ Use of lithium at screening significantly different between the groups (χ^2^ = 6.23, Fisher’s exact *p* =. 049)

Of the 102 randomized patients, 81 finished the ESM protocol (i.e., completed the ESM post-assessment). Treatment assignment was not associated with probability of finishing the ESM study protocol (χ^2^ (2) = 1.49, *p* =. 47) and number of attended intervention sessions did not differ between the experimental and pseudo-experimental groups (F (1, 68) = 1.66, p =. 20).

Previous work has shown that ESM assessments with less than 30% of filled out beeps are less reliable and consequently not valid [14]. Therefore, ESM periods (one period corresponding to one ESM ‘week’ in the protocol) with less than 30% of beeps completed were excluded from the analyses, leading to the exclusion of one baseline ESM period, four post ESM periods, and two periods during the intervention period (Fig 1).

### ESM measures and experimental sessions

**Fig 2:**
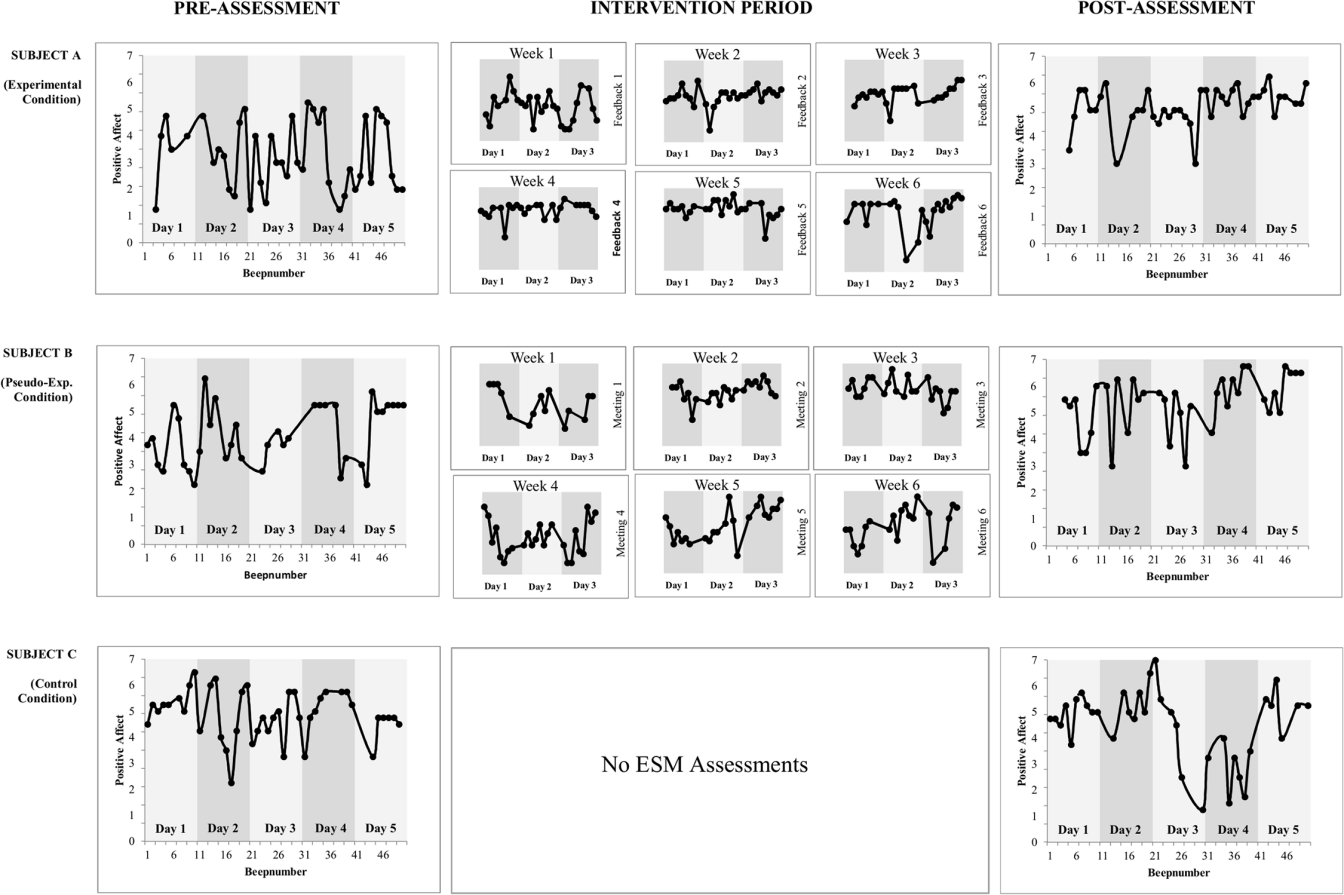
Individual PA series across the study protocol for three participants either randomized into the experimental (subject A), pseudo-experimental (subject B) or control group (subject C). Dots represent the average momentary ratings on the adjectives composing PA (cheerful, enthusiastic, relaxed, satisfied) during one beep. One day comprises ten beeps; pre- and post-assessment comprises five days; the intervention period comprises three days of ESM monitoring per week.

Participants completed 16,678 device entries in total. Of these, 83 observations (<1%) were excluded as invalid because less than 30% of beeps for that ESM period were filled in. On average, participants responded to 39.7 (out of maximal 50) beeps during the 5-day ESM periods (pre-and post- assessment) and to 23.7 (out of maximal 30) during the 3-day periods (intervention). Fig 2 shows the individual PA series across the study protocol for one participant of each condition.

Feedback sessions lasted on average 48.9 minutes (SD 11.2, range 27–105 minutes), which was significantly longer (b = 9.57, p<.001) than the duration of pseudo-experimental interview sessions (mean: 39.5, SD 12.9, range 15–90 minutes).

Pre-post change in positive affect (χ^2^(3) = 0.48, *p* =. 923) or negative affect (χ^2^(3) = 0.57, *p =*. 904) was not associated with the person who conducted the assessment or delivered the (pseudo-) intervention.

### Effects of the intervention on positive and negative affect from pre- to post-assessment

#### Positive affect

The mean for positive affect increased by 0.3 points in the experimental group, by 0.1 points in the pseudo-experimental group, and in the control group by 0.2 points (see Table 1). Although the experimental group showed the largest increase in PA at mean level, there was no significant interaction between group (experimental, pseudo-experimental, control group) and time (pre- vs. post-assessment) (χ^2^(2) = 0.33, *p* =. 846)(Table 2).

**Table 2:** Results of mixed effects models: Change in affect from pre to post-assessment.

Variable	Positive affect	Negative affect
	**b (SE); [CI]**	**b (CI); [CI]**
**Group** ^**a**^		
Pseudo-exp.	0.17 (0.22);[-0.26, 0.59]	-0.07 (0.24); [-0.54, 0.39]
Exp.	0.21 (0.22); [-0.23, 0.64]	-0.02 (0.24); [-0.49, 0.45]
**Time** ^**b**^	0.15 (0.16); [-0.16, 0.46]	0.19 (0.12); [-0.04, 0.43]
Group*Time		
Pseudo-exp./post assessment	-0.01 (0.21); [-0.43, 0.41]	-0.41 (0.16); [-0.73, -0.09]^c^
Exp./post-assessment	-0.11 (0.22); [-0.55, 0.33]	-0.23 (0.17); [-0.56, 0.10]
	**Estimation (SE)**	**Estimation (SE)**
**Random Effects**		
Intercept SD (Participant)	0.89 (0.06); [0.77, 1.03]	0.97 (0.07); [0.84, 1.12]
Slope SD (Time)	0.11 (0.01); [0.09, 0.13]	0.08 (0.01); [0.07, 0.10]

b = Regression coefficient; SE = Standard Error; SD = Standard Deviation; CI = Confidence Interval; Pseudo-exp. = Pseudo-experimental group; Exp. = Experimental group

^a^ Reference category: Control group

^b^ Reference category: Pre-assessment

^c^ *p* = 0.012

#### Negative affect

Table 2 shows the detailed results of the mixed effects model. In contrast to PA, there was a significant interaction between group (experimental, pseudo-experimental, and control group) and time (pre- vs. post-assessment) (χ2(2) = 6.29, *p* =. 043). Adjusted predictions showed that the control group was associated with an increase of NA from 2.28 to 2.47; the pseudo-experimental group was associated with a decrease of NA from 2.21 to 1.99; the experimental group was associated with a decrease of NA from 2.26 to 2.22. The slopes between the pseudo-experimental group and control group differed significantly (b = -0.40, χ^2^(1) = 6.29, *p* =. 012), denoting that while the pseudo-experimental group decreased in NA by 0.19 points on a scale from 1 to 7, the control group increased by 0.21 points. There was no significant difference in slopes between participants in the pseudo-experimental group and the experimental group (b = -0.22, χ^2^(1) = 1.17, *p* =. 279), or between participants in the experimental group and the control group (b = -0.20, χ^2^(1) = 1.85, *p* =. 174).

### Effects of the intervention on positive and negative affect over the course of the intervention

**Fig 3:**
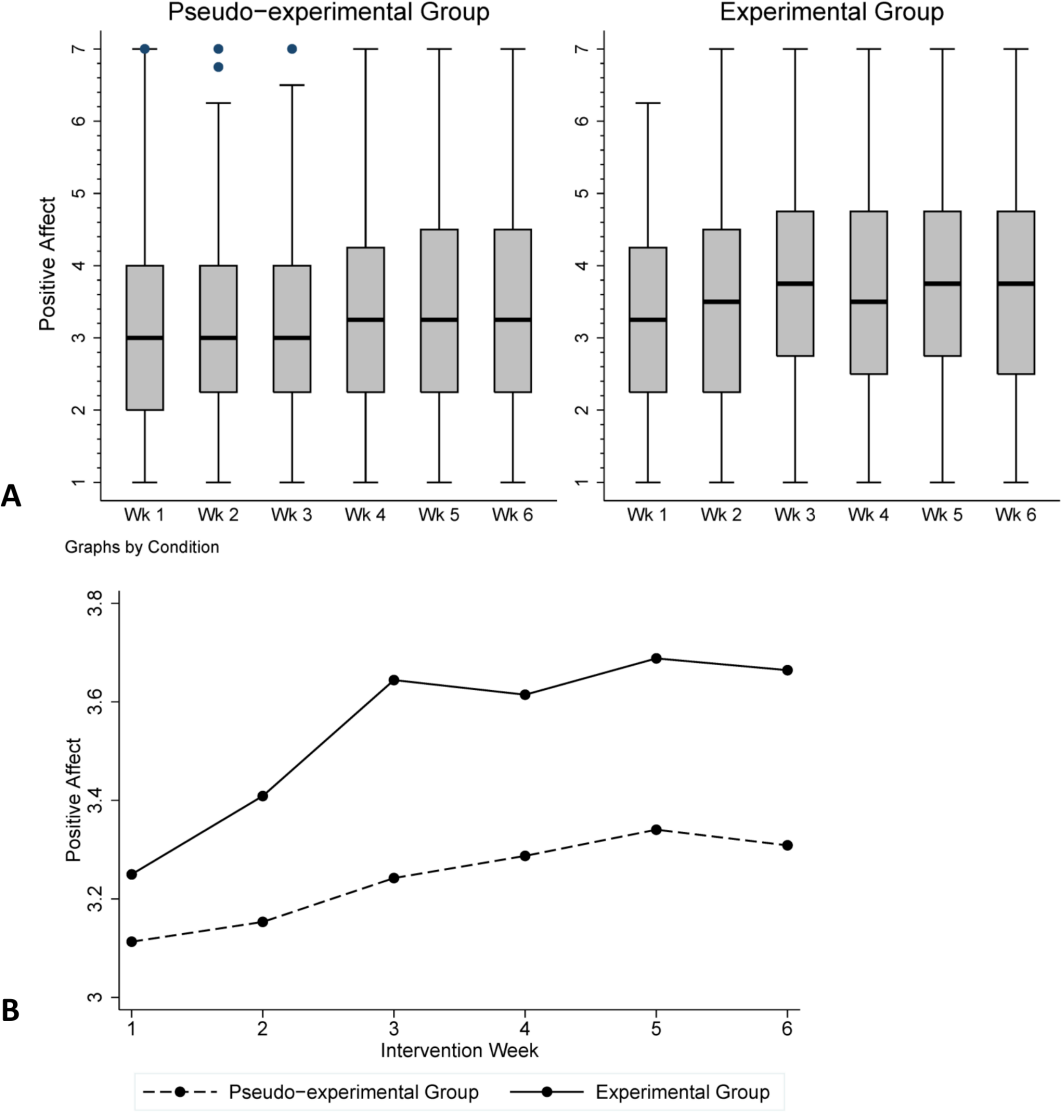
Part A: Box-Whisker plot of PA across the intervention period, by condition. The bottom and top of box indicate first (Q1) and third quartile (Q3), the band indicates the median; whiskers represent upper and lower adjacent values (within Q3+1.5(Q3-Q1) and Q1-1.5(Q3-Q1), respectively. Part B: Means per week across intervention period, by condition.

Fig 3 shows box-whisker diagrams and means for PA over the course of the intervention period for the experimental and pseudo-experimental group.

Statistical modelling yielded no significant interaction between treatment allocation and intervention week (1 to 6) (χ^2^(5) = 0.24, *p* =. 999) for positive affect. Likewise, there was no significant interaction between treatment allocation and intervention week (1–6) for negative affect (χ^2^(5) =. 50, *p* =. 992). See Table 3 for the detailed results of the mixed effects model.

**Table 3:** Results of mixed effects models: Change in affect during intervention period.

Variable	Positive affect	Negative affect
	**b (SE); [CI]**	**b (CI); [CI]**
**Group** ^**a**^	0.14 (0.29), [-0.43, 0.70]	0.16 (0.27), [-0.36, 0.68]
**Time** ^**b**^		
Week 2	0.10 (0.28); [-0.46, 0.65]	>-0.01(0.26); [-0.51, 0.51]
Week 3	0.16 (0.28); [-0.39, 0.72]	-0.04 (0.26); [-0.55, 0.47]
Week 4	0.20 (0.29); [-0.36, 0.76]	-0.22 (0.26); [-0.73, 0.30]
Week 5	0.24 (0.29); [-0.33, 0.80]	-0.04 (0.26); [-0.55, 0.48]
Week 6	0.21(0.29); [-0.35, 0.78]	-0.23 (0.27); [-0.75, 0.28]
Group*Time		
Exp./week 2	0.03 (0.41); [-0.78, 0.84]	-0.14 (0.38); [-0.88, 0.60]
Exp./week 3	0.13 (0.42); [-0.68, 0.95]	-0.14 (0.38); [-0.89, 0.60]
Exp./week 4	0.09 (0.42); [-0.73, 0.91]	-0.04 (0.38); [-0.79, 0.71]
Exp./week 5	0.12 (0.42); [-0.71, 0.94]	-0.24 (0.39); [-0.10, 0.51]
Exp./week 6	0.17 (0.42); [-0.66, 1.00]	-0.08 (0.39); [-0.84, 0.68]
	**Estimation (SE)**	**Estimation (SE)**
**Random Effects**		
Slope SD (Week)^c^	1.17 (0.04); [1.09, 1.26]	1.08 (0.04); [1.01, 1.16]

b = Regression coefficient; SE = Standard Error; SD = Standard Deviation; CI = Confidence Interval; Pseudo-exp. = Pseudo-experimental group; Exp. = Experimental group

^a^ Reference category: Pseudo-experimental group

^b^ Reference category: Week 1

^c^ Intercept (subject) suppressed

## Discussion

Building on evidence that PA may be a crucial system involved in phenotypic resilience in depression [7, 12] and capitalizing on recent technical developments making electronically collected ESM data readily available [17, 18], the present RCT focused on intervening at the level of positive affective experience in daily life by providing PA-focused feedback derived from self-collected ESM data (ESM-I). In an earlier analysis in the same sample, we showed that ESM-I was associated with a clinically relevant reduction of depressive symptoms commencing three weeks after the intervention [20]. In the present study, we zoomed in on the micro-level of every-day positive and negative affective experiences *during* the intervention, possibly shedding light on the mechanisms through which ESM-I may occasion a reduction in depressive symptomatology at the macro-level. That is, it was investigated if the PA-focused ESM-I was indeed associated with an increase in positive affective experience in daily life during and shortly after the intervention.

Multilevel regression analysis yielded no statistically significant differences in the change from pre- to post-test for the three different conditions. Likewise, the analysis yielded no statistically significant differences in PA *during* the intervention period between the experimental and pseudo-experimental group. An inspection of the random effects in the mixed effects model suggests rather large individual differences. As such, it may be the case that the effect of the intervention on PA is highly individual specific. Alternatively, the nonsignificant findings may relate to the period chosen during which ESM monitoring took place. Based on our previous report, the differences in depressive symptoms between the experimental group and control group became apparent three weeks after completion of the intervention and the differences grew stronger over time. However, ESM data on momentary PA was, due to patient burden, only available in the period during- and shortly (one week) after the intervention. It is possible that an increase in positive affective experience would only become visible *after* the measurement window of one week post-intervention. According to the broaden-and-build theory [30], experiencing PA may increase the focus of an individual, enabling him or her to pay more attention to rewarding events and to augment engagement in PA evoking activities, leading to the experience of even more positive affect. It may be that the development of such an emotional “upward spiral” may take some time to develop. The translation of receiving PA-focused feedback in the clinical setting to the actual exploration of PA-evoking activities in daily life, and eventually being able to activate the “upward spiral” of positive emotions, is probably a process of trial and error, which may take some time to evolve. It is possible that this process occurred after our ESM sampling window and therefore a change in affect was not captured (see also the limitations section). Alternatively, the question may arise if the ESM-based PA and NA were measured too momentarily to actually be able to pick up differences. However, this possibility seems unlikely as previous studies using similar designs and identical PA and NA assessments were able to show changes in momentary PA after an intervention [7, 12, 31]. Finally, an alternative possibility is that an increase in momentary PA is not the mechanism behind the observed reduction in depressive symptoms in the experimental group and that it is necessary to search for alternative mechanisms. Promising future directions for this search include investigating the temporal dynamics of affect (i.e. variability/temporal dependency) and conducting within-person mediation analyses using multilevel path analyses.

While the results indicated no significant change in NA levels in the experimental group during the intervention period, participants in the pseudo-experimental group showed a stronger decrease in NA from pre- to post-assessment compared to the control group. This is in line with our preceding paper, demonstrating that the pseudo-experimental group showed a reduction in depressive symptoms directly during the intervention period; however, this effect was not persistent as depressive symptoms in the pseudo-experimental group started to increase again during the follow-up period [20]. The decrease in NA may reflect a non-specific placebo response as patients may have thought that they were receiving the experimental intervention. Alternatively, the sessions with the researcher, during which the patient was able to talk about his or her depressive feelings during the previous week, may have resulted in the specific reduction of NA. Instead, it may be that merely the monitoring of affective responses, behaviour and context in daily life for the duration of six weeks may be associated with increased momentary emotional awareness, making ESM interesting for use in mindfulness-based therapies [32].

### Limitations

The present RCT did not include a fourth treatment arm, that is, a pseudo-experimental group consisting of a six-week period of ESM self-monitoring only, without weekly contacts with the researcher. Therefore, it was not possible to separate the effect of self-monitoring from the effects of weekly contacts with the researcher. This treatment arm was not included since the primary goal of the present RCT was to investigate the effects of feedback based on ESM, and not the effects of ESM self-monitoring per se.

ESM was only performed over the course of the intervention and not during the 6-month follow-up periods. As such, data with respect to daily life momentary PA and NA were only available for the duration of the intervention period and the moments immediate before and after. In light of our previous results with regard to the effects of ESM-I on depressive symptoms, showing that the effect of ESM-I may take some weeks to evolve, it would have been instructive to also have ESM data available in the weeks after completion of the intervention. This would potentially have shed more light on the micro-mechanisms by which ESM-I did reduce depressive symptoms in the long term. It is a challenge for future ESM studies to identify the ideal time frames for ESM monitoring in order to fully investigate the mechanisms of interventions at the micro-level while minimizing patient burden.

Finally, a considerably high number of patients who were initially contacted declined to participate in this rather demanding trial. As such, it is possible that the present patient population presented with certain (unassessed) patient characteristics.

## Conclusion

In the present trial, we showed that a novel intervention based on ESM (referred to as ESM-I), consisting of weekly feedback on the experience of PA in daily life, did not significantly impact momentary PA during or shortly after the intervention. This finding contrasted with previous results in this sample, demonstrating that the PA-focused ESM-I was associated with a decrease in depressive symptoms on the long-term. As this reduction in depressive symptoms only unveiled itself after weeks, it is conceivable that the effects of the feedback on momentary PA also evolved slowly and therefore were not captured by the experience sampling procedure during and immediately after treatment. In light of these findings, an extension of the ESM measurement window to follow-up measures when evaluating ESM-I, is recommended. Furthermore, given these inconsistent findings and novelty of ESM-I, additional studies are needed to establish effectiveness and optimal ESM-I content.

## Supporting Information

S1 CONSORT Checklist CONSORT 2010 checklist. (DOCX)

S1 Dataset Relevant dataset excluding personal information. (XLS)

S1 Feedback Example of the graphical feedback with verbal information (extract). (DOCX)

S1 Protocol Original study protocol. (PDF)
