# Supplementary material for: Experience Sampling-Based Personalized Feedback and Positive Affect: A Randomized Controlled Trial in Depressed Patients
Source: PLoS One. 2015 Jun 2;10(6):e0128095. doi: 10.1371/journal.pone.0128095 (PMC4452775; doi:10.1371/journal.pone.0128095)
Supplement: S1 Feedback — (DOCX) [file pone.0128095.s003.docx]

Feedback S1. Example of the graphical feedback with verbal information (extract).

‘I will now give you feedback about the data from the last week. This feedback refers to the moments that you filled in the PsyMate beep questionnaire. This is mainly about how often you experienced positive feelings, and in which context that was. Positive feelings are namely important in the recovery from depression. Furthermore, the experience of positive feelings is important to prevent a relapse into a new depression. This has been shown by scientific research. The goal of this training is to give you more insight into your feelings. We also look into the circumstances in which you experience these feelings. You can use this knowledge to learn to regulate your feelings and increase your emotional resilience. I will give the feedback through charts which I will discuss with you. You can take these charts home if you like. You will also receive a letter at your home with a summary of the most important points of today.

Do you have any questions?

[…]

Here [participant is shown type of activities chart above], you can see what you have done during the last week (during beep moments). As you can see, you spent most of your time at work, engaging in housekeeping, talking with others, and doing nothing or resting. You have spent a little time on passive relaxation and active relaxation. The least frequent activities (during beep moments) were grooming and being en route. Is this clear to you? Do you recognize this? Were you aware of this?

We’ve also had a look at your activities and your feelings during these activities. In this graph [participant is shown positive feelings graph above], we can see the link between the type of activity and the degree of positive feelings you experienced during this activity. The further a bar reaches to the right, the more positive you felt during that particular activity. Let’s have first a look into the relaxation/doing nothing activities. As you can see, you reported most positive feelings during active relaxation, a bit less during passive relaxation and the least positive feelings during nothing/resting (compared to the other two activities). Considering all activities, you had most positive feelings during active relaxation, followed by passive relaxation, grooming, and talking with others. You experienced least positive feelings during work/study, and household activities. If we look back to the ‘type of activity’ graph [participant is shown type of activity graph again], we can see that although active relaxation was the activity which was linked to most positive feelings, it is almost the least frequent activity.

Is this clear to you? Do you recognize this? Were you aware of this?

[…]

This graph shows the degree of positive feelings per week. One bar corresponds to one week. As we can see here [participant is shown degree of positive feelings per week], your positive feelings seem to increase. This week (as well as in week 4 and 5) you had on average a 5. If we compare that to the baseline and week 1, you had on average a 3.

Is this clear to you? Do you recognize this? Were you aware of this?

[…]
