# Supplementary material for: Experience Sampling-Based Personalized Feedback and Positive Affect: A Randomized Controlled Trial in Depressed Patients
Source: PLoS One. 2015 Jun 2;10(6):e0128095. doi: 10.1371/journal.pone.0128095 (PMC4452775; doi:10.1371/journal.pone.0128095)
Supplement: S1 Protocol — (PDF) [file pone.0128095.s004.pdf]

Research protocol

(July 2009)

PROTOCOL TITLE 'Active mobilisation of psychological resources during passive antidepressant pharmacotherapy'

**Protocol ID**	**Active mobilisation of psychological resources during passive antidepressant pharmacotherapy**
**Short title**	**REsource MObilisation Device In Depression (REMOD-ID)**
**Version**	**1**
**Date**	**July 2009**
**Coordinating investigator/project leader**	**J.J. Van Os, head of department Psychiatry & Neuropsychology, University of Maastricht, the Netherlands**
**Principal investigator(s) (in Dutch: hoofdonderzoeker/uitvoerder)**<br>***Multicenter research: per site***	**M.C. Wichers, department Psychiatry & Neuropsychology, University of Maastricht**<br>**Tel: 043-3688669**
**Sponsor (in Dutch: verrichter/opdrachtgever)**	**J.J. Van Os, head of department Psychiatry & Neuropsychology, University of Maastricht**
**Independent physician(s)**	**M. Bak, department Psychiatry & Neuropsychology, University of Maastricht**<br>**Tel: 043-3688669**
**Laboratory sites <*if applicable*>**	**Not applicable**
**Pharmacy <*if applicable*>**	**Not applicable**

PROTOCOL SIGNATURE SHEET

Name	Signature	Date
**Sponsor or legal representative:**<br>***<please include name and function>***<br><br>**For non-commercial research,**<br>**Head of Department:**<br>***<include name and function>***	**J.J. Van Os, head of department Psychiatry & Neuropsychology, University of Maastricht, the Netherlands**	**15-07-2009**
**Coordinating Investigator/Project leader/Principal Investigator:**<br>***<please include name and function>***	**J.J. Van Os, head of department Psychiatry & Neuropsychology, University of Maastricht, the Netherlands**<br><br>**M.C. Wichers, department Psychiatry & Neuropsychology, University of Maastricht, the Netherlands**	**15-07-2009**

TABLE OF CONTENTS

1. INTRODUCTION AND RATIONALE 9
2. OBJECTIVES 12
3. STUDY DESIGN 13
4. STUDY POPULATION 18
4.1 Population (base) 18
4.2 Inclusion criteria 18
4.3 Exclusion criteria 18
4.4 Sample size calculation 18
5. TREATMENT OF SUBJECTS 19
5.1 Investigational product/treatment 19
5.2 Use of co-intervention (if applicable) 19
5.3 Escape medication (if applicable) 19
6. INVESTIGATIONAL MEDICINAL PRODUCT 19
7. METHODS 20
7.1 Study parameters/endpoints 20
7.1.1 Main study parameter/endpoint 20
7.1.2 Secondary study parameters/endpoints (if applicable) 20
7.1.3 Other study parameters (if applicable) 21
7.2 Randomisation, blinding and treatment allocation 23
7.3 Study procedures 23
7.4 Withdrawal of individual subjects 24
7.4.1 Specific criteria for withdrawal (if applicable) 24
7.5 Replacement of individual subjects after withdrawal 25
7.6 Follow-up of subjects withdrawn from treatment 25
7.7 Premature termination of the study 25
8. SAFETY REPORTING 25
9. STATISTICAL ANALYSIS 26
9.1 Descriptive statistics 26
9.2 Univariate analysis 26
9.3 Multivariate analysis 27
9.4 Interim analysis (if applicable) 28
10. ETHICAL CONSIDERATIONS 29
10.1 Regulation statement 29
10.2 Recruitment and consent 29
10.3 Objection by minors or incapacitated subjects (if applicable) 29
10.4 Benefits and risks assessment, group relatedness 29
10.5 Compensation for injury 30
10.6 Incentives (if applicable) 30
11. .ADMINISTRATIVE ASPECTS AND PUBLICATION 32
11.1 Handling and storage of data and documents 32
11.2 Amendments 32

11.3 Annual progress report 32
11.4 End of study report 32
11.5 Public disclosure and publication policy 33
12. REFERENCES 33

LIST OF ABBREVIATIONS AND RELEVANT DEFINITIONS

**ABR**	**ABR form (General Assessment and Registration form) is the application form that is required for submission to the accredited Ethics Committee (ABR = Algemene Beoordeling en Registratie)**
**AE**	**Adverse Event**
**AR**	**Adverse Reaction**
**CA**	**Competent Authority**
**CCMO**	**Central Committee on Research Involving Human Subjects**
**CV**	**Curriculum Vitae**
**DSMB**	**Data Safety Monitoring Board**
**EU**	**European Union**
**EudraCT**	**European drug regulatory affairs Clinical Trials GCP Good Clinical Practice**
**IB**	**Investigator's Brochure**
**IC**	**Informed Consent**
**IMP**	**Investigational Medicinal Product**
**IMPD**	**Investigational Medicinal Product Dossier**
**METC**	**Medical research ethics committee (MREC); in Dutch: medisch ethische toetsing commissie (METC)**
**(S)AE**	**Serious Adverse Event**
**SPC**	**Summary of Product Characteristics (in Dutch: officiële productinfomatie IB1-tekst)**
**Sponsor**	**The sponsor is the party that commissions the organisation or performance of the research, for example a pharmaceutical company, academic hospital, scientific organisation or investigator. A party that provides funding for a study but does not commission it is not regarded as the sponsor, but referred to as a subsidising party.**
**SUSAR**	**Suspected Unexpected Serious Adverse Reaction**
**Wbp**	**Personal Data Protection Act (in Dutch: Wet Bescherming Persoonsgevens)**
**WMO**	**Medical Research Involving Human Subjects Act (Wet Medisch-wetenschappelijk Onderzoek met Mensen**

SUMMARY

Rationale: Recent evidence indicates that individual daily life person-context interactions determine vulnerability for depression, and predict relapse as well as recovery. We hypothesize that momentary assessment technology (the MET-D device) to monitor these daily life person-context interactions during treatment will enrich passive antidepressant pharmacotherapy with an active resource-mobilising therapeutic context, thus enhancing therapeutic efficacy (reducing symptoms and relapse rate). Genetic factors play an important role in depression and sensitivity to stress and may therefore impact on the therapeutic efficacy of monitoring with MET-D.

Objective:

(1) Does feedback on continuous monitoring with MET-D during pharmacotherapy result in a better treatment response (immediate and follow-up reductions of depressive symptoms, decreased relapse risk)?

(2) Is feedback on continuous monitoring with MET-D during pharmacotherapy cost-effective?

(3) Do measurements of daily life person-context interactions improve the prediction of future courses of depressive symptoms?

(4) Can individual differences in (1) the effect of feedback on daily life person-context interaction and (2) the predictive value of these person-context interactions be traced back to genetic differences?

Study design: Randomized clinical trial with three groups of 40 subjects each (2 experimental groups and 1 control group). The first group receives a 5-day pre- and post MET-D assessment *and* a continuous MET-D assessment (3 days of MET-D measurements during a 6-weeks period) *with weekly feedback* (to both patient and therapist) during treatment as usual (TAU). The second group also receives a 5-day pre- and post MET-D assessment *and* a continuous MET-D assessment (3 days of MET-D measurements during a 6-weeks period) but *without feedback* during treatment as usual (TAU). The third group receives a 5-day pre- and post MET-D assessment but no additional intervention during TAU. This is the control group.

Study population: A sample of patients (n=120) with a depressive disorder will be recruited within (i) SEARCH, the research network of mental health institutions within Limburg and South-Brabant, The Netherlands, (ii) general practices and (iii) the general population.

Intervention (if applicable): The MET-D is a recently developed wearable interactive palmtop suitable for the Experienced Sampling Method (ESM) to study subjects in their daily life. The intervention group receives feedback on MET-D measurements of reward experience in daily life.

Main study parameters/endpoints: The rate of change in reward experience measured with the MET-D, and the change in depression symptomatology measured with the Structured Clinical Interview for DSM-IV Axis I Disorders (SCID-I) and the Hamilton Depression Rating

Scale (HDRS). Reward experience is conceptualised as the degree of positive affect following small positive daily events.

Nature and extent of the burden and risks associated with participation, benefit and group relatedness: There are no health risks associated with participation. Personal benefits for the subjects are (i) an increase in insight and control over recovery mechanisms, and (ii) the standard treatment can be adapted to the individual needs with the use of feedback. Burden for the patients is investment of time. After a screening procedure (3 hours), the time investment for subjects is approximately 5.5 hours comprising interviewing and self-report questionnaires (distributed over 7 meetings). In addition, all subjects have to participate twice in a 5-days MET-D measurement period (approximately 4 hours in total). Time investment for subjects in both experimental groups comprises an additional 13.5 hours. MET-D measurement takes place in the living environment of the patient, and as such reduces the burden for the patient.

1. INTRODUCTION AND RATIONALE

According to the World Health Organisation (WHO), depression is among the leading causes of disability worldwide, affecting about 121 million people worldwide of whom fewer than 25% have access to effective treatment. A disorder which is so prevalent conveys a very large burden on the health care system and results in large accompanying costs of health care and loss of productivity in the economic sector. In the Netherlands, depression has a life time prevalence of 15.4%, and a 12-month prevalence of 5.8% (NEMESIS-study) [1]. Residual symptoms after antidepressant therapy, relapse and treatment-resistance are often observed. The risk for chronicity is considerable (20%) [2].

Recently evidence is emerging that daily life person-context interactions not only determine vulnerability for depression [3], but also predict recovery [4] as well as relapse [5]. This creates an interesting window of opportunity, not only for the study of the etiology of depression, but also for the study of intervention possibilities. Daily person-context interactions involve the way a person reacts to his or her environment. For example, a telephone call with an unsatisfied client making you feel tensed for the rest of the day. Depression is associated with low positive affect (anhedonia) and with high negative affect. These are separate and independent affect dimensions [6]. The degree of positive affect following small positive daily events is known as the concept of 'reward experience'. Depressed persons experience a low level of reward during daily life. Reward experience is important because it can buffer negative affect in depressive subjects. One study showed that recovery in depression occurred in those with increases in the ability to experience reward from daily life situations during antidepressant treatment[4]. Furthermore, positive emotions in daily life make people more resilient against depression because it reduces the relapse rate for depressive episodes [5]. For example, a pilot study (n=61) was performed that examined whether affective response patterns in daily life influenced future relapse in individuals with a history of depression. The data showed that positive affective responses to pleasant events (reward experience) were an important predictor of future reduction in depressive symptoms and relapse. These studies indicate that (i) improvement of reward experience is likely to aid recovery and reduce future relapse and is thus an attractive target for the study of intervention possibilities and (ii) that information from individual affective responses to daily life contexts provides extra information that may help improved prediction of future course of depression.

In the field of depression a large number of studies examined the relationship between environmental stressors (such as negative life events) and the development of depressive symptoms [7]. Results suggest that although the existence of negative life events is an important risk factor in the development of depression, the existence of minor stressors in daily life may have an even larger effect due to its daily repetitive and possibly cumulative nature [8]. Daily

negative events result in the development of large amounts of negative affect which lead to a high risk for development of depressive symptoms [3].The degree of negative affect following small negative daily events is known as 'stress sensitivity' [3]. Earlier studies have shown that stress sensitivity is an underlying mechanism with a genetic basis in the etiology of depression [3], and as such can be regarded as an endophenotype of depression [9].

The measurement of reward experience and stress sensitivity as operationalisations of daily person-context interactions creates an opportunity to zoom in on personal coping mechanisms, examining the way a subject interacts with his of her environment. This can on the one hand result in knowledge of the way a person enables his personal resources in coping with depressive symptoms, and on the other hand result in better monitoring compared to usual measures of treatment efficacy [10].

Measurement of daily person-context interactions must take place at those moments in which an individual interacts with his environment to provide ecological valid information. The Experience Sampling Method (ESM) is suitable for this purpose [11-13]. Several studies of depression have been performed with the ESM paradigm, and this procedure is not only ecologically valid but proves to have a good validity and reliability [14-16]. The validity and reliability of ESM for the measurement of direct effects of daily stressors on mood has also been demonstrated [3, 11-13]. New technology to electronically monitor the patients' dynamic daily life responses to their personal context for extended periods (4-6 weeks) has recently become available and is patented by our group under the name 'MET-D device'. This device is not only suitable for ESM, but it can also electronically summarize the information of daily person-context interactions. This creates the possibility to provide feedback to subjects on their patterns of emotional responses to daily life situations. Receiving feedback on behavior can result in emotional and behavioral change, as is already known from the field of behavioral therapy [17-20]. Feedback on MET-D measurements of reward experience and affect variability may create an active and possibly therapeutic context for the depressed patient. Feedback can be given to both patient and therapist. For the patient, feedback can result in an increased insight in personal functioning, awareness of small improvements leading to more control over possible causes of depressed mood, and finally an increased use of the patient's personal resources. For the therapist, feedback can result in a better monitoring of both compliance and the effect of the treatment. The application of feedback on dynamic daily life person-context interactions, as measured with the MET-D, may improve therapeutic efficacy by means of enrichment of passive antidepressant pharmacotherapy with an active resource-mobilizing therapeutic context.

The aim of this study is to examine the therapeutic and predictive possibilities of the MET-D. We expect that (i) MET-D feedback on reward experience and affect variation enhances pharmacotherapeutic efficacy resulting in a better treatment response (immediate and follow-up

reductions of depressive symptoms and reductions in relapses) leading to a reduction in health care consumption and costs.

A second aim of this study is to evaluate whether the use of the MET-D, from the viewpoint of the society, is preferable compared to Usual Care in terms of effects (depressive symptoms, relapse), utilities (quality of life), and costs. Based on this main research question several sub-questions are relevant, such as: 1a) What are the (extra) costs of the delivery of MET-D (with and without feedback) compared to Usual Care? 1b) What are the (extra) effects (measured in depressive symptoms, relapse, and quality of life) of MET-D (with and without feedback) compared to Usual Care? We expect that (ii) MET–D feedback on reward experience and affect variation will be cost-effective.

Furthermore, we hope to confirm recent results regarding the role of reward experience, as described above, in the prediction of a depressive relapse [5]. We expect that (iii) the ability to generate reward experience predicts a decreased risk for the development of a depressive relapse. MET-D measurement of reward experience may thus lead to an improvement of the prediction of future courses of depressive symptoms.

Not only person-context interactions, as measured with the MET-D, are important in the etiology of depression and in the prediction of future depressive episodes. Evidence on the important role of genetic influences on both depression as well as on stress sensitivity is emerging [3, 21-27]. For example, subjects who are ‘Met’ carriers of the BDNF gene exhibit increased stress sensitivity compared to the ‘Val/Val’ carriers [28]. Since stress sensitivity influences treatment response [4], it can be hypothesized that treatment effect may be partly under genetic control, as is demonstrated by recent findings [29]. This may be due to the intermediate action of stress sensitivity. Therefore, our final goal is (iiii) to examine the influence of genetic differences on both the effect of MET-D feedback on treatment response, and on the predictive value of daily life person-context interactions on relapse risk.

2. OBJECTIVES

The objectives are to examine in a randomized controlled trial whether:

1) feedback on relevant daily life person-context interactions (as measured with MET-D) during pharmacotherapy directly affects treatment response in terms of immediate and follow-up decrease of depressive symptomatology and relapse

2) feedback on relevant daily life person-context interactions (as measured with MET-D) during pharmacotherapy is cost-effective

3) measurement of relevant dynamic daily life behaviours significantly contributes to the prediction of the future course of depressive symptomatology.

4) individual differences in (i) the effect of feedback on daily life person-context interaction and (ii) the predictive value of these person-context interactions can be traced back to genetic differences

3. STUDY DESIGN

Experience Sampling Method (ESM):

ESM is a structured diary method developed to assess subjects in their daily surroundings. This method has been validated for the use of studying the immediate effects of stressors on mood [11, 14, 16]. Most ESM studies use the paper-and-pencil method in answering and registering the ESM questions. Recently our research group has patented an interactive ESM palmtop (the MET-D). The feasibility and validity of computerized ESM has been demonstrated [30]. The MET-D signals subjects at random moments of the day to answer MET-D produced questions about affect and daily events. Answering the MET-D questions after each auditory signal (“beep”) will take about 30 seconds, which is far less demanding for the subjects compared to the traditional paper-and-pencil ESM method. The MET-D is programmed to emit 10 times a day a beep at random intervals in each of the ten 90-minutes time blocks between 7:30h and 22:30h, on 5 consecutive days during pre- and post-intervention measurements. Additionally, for the MET-D intervention group, the MET-D will be programmed to emit 10 times a day a beep at random intervals in each of the ten 90-minutes time blocks between 7:30h and 22:30h on 3 consecutive days a week during a 6-week period. After each beep, subjects have to fill out the self-assessment on the MET-D device to record current context (activity, persons present, location, physical activity), stress appraisals of this context, and mood. Mood questions include 4 Positive and 5 Negative Affect items. Examples are ‘happy’ and ‘relaxed’ for positive affect and ‘depressed’ and ‘irritated’ for negative affect. The self-assessments will be rated on 7-point Likert scales (ranging from 1= ‘not at all’ to 7= ‘very’). Subjects will be instructed to complete the MET-D measurements as quickly as possible after the beep. This emphasis helps to minimize retrospective memory distortion.

Design:

A randomized controlled trial will be conducted with three treatment arms:

- The **first** arm (‘MET-D intervention *with* feedback’; n=40) receives 5-day pre- and post MET-D assessment *and* continuous MET-D assessment (each week 3-days MET-D measurement during a 6- weeks period) *with feedback* (to both patient and therapist) during treatment as usual (TAU= antidepressant pharmacotherapy). This is the experimental group.

- The **second** arm (‘MET-D intervention *without* feedback’; n=40) also receives a 5-day pre- and post MET-D assessment *and* a continuous MET-D assessment (3 days of MET-D measurements during a 6-weeks period) but *without feedback* during treatment as usual (TAU). In order to prevent any effects of different duration of the weekly contacts with the researcher, alternative weekly activities (interviews on anxiety and depression) are carried out. This is the control experimental group.

- The **third** arm (control group; n= 40) receives 5-day pre- and post MET-D assessment but no additional intervention during TAU.

Feedback:

Patients in the first arm receive MET-D feedback after each intervention week. This sums up to a total of 6 feedback moments. The MET-D feedback will be given verbally, written, and graphically (in clear pie charts and bar graphs) to both patient and therapist, showing emotional responses to daily life activities, events and social situations and how these daily life situations relate to momentary affective responses of the participant. The researcher will communicate the feedback verbally, according to a standardized protocol, to the patient. The researcher gives the same feedback to the treating therapist (if applicable). The therapist can additionally support the participant in modifying his/her daily life behaviour towards situations of more positive emotions and adjust therapeutic strategy or advice. Patient and treating therapist will receive a written copy of the feedback from the researcher. The feedback will focus on change in positive emotional responses in daily life in order to refocus and redirect the participant towards occasions for emotional strength. The feedback shows the participant's changes in affective responses in daily life situations since the start of the study. In addition, the feedback will show the relationship between (change in) affective responses in daily life and current depressive symptoms. In this way, the patient gets increased control over the cause of his/her depression and is made aware of small improvements.

Examples of what the participant will know after a feedback session:

-The average amount of his/her physical activity in the past days. How it changed compared to the last measurements (did it increase or decrease?). What the relation is between his/her moments of physical activity and the positive emotions generated at those moments. The data may show that the participants receive more positive emotions in moments of more physical activity than less physical activity. The researcher communicates this information in such a way that the participant understands what it means and how it relates to his/her daily life behaviour. Furthermore, the researcher mentions the way the participant can change his/her daily life activities in a way that would lead to more positive emotions ('if you want to train your emotional strength you can try to do more regular physical exercise').

-The average amount of time spent in different kinds of company (partner, friends, colleagues, family, strangers, or alone) in the past days. How the type of company relates to positive emotions generated at that moment. How well the participant can generate positive emotions from social situations he/she rated as pleasant (reward experience) and how this ability changed over time. Again, the researcher communicates the information in understandable language and provides compact advice on how to change behaviour associated with increases in positive

emotions. For example, ‘you say you do not feel like meeting people, but your own daily life recordings show that when you are in company you, experience more positive emotions than when alone. Therefore, if you want to train your emotional strength you can try meeting more often with people you like’.
The researcher gives the same feedback to the treating therapist (if applicable). The therapist can additionally support the participant in modifying his/her daily life behaviour towards situations of more positive emotions and adjust therapeutic strategy or advice.

Although the participants respond to the same questions, generated by MET-D, throughout the intervention period, the feedback is built up in three modules:
The first sessions (1 and 2) are focused on daily life activities.
Sessions 3 and 4 are focused on daily life events and how patients deal with things that happen in daily life. In addition, at the end, patients also receive information on their progress in daily life activities as discussed in sessions 1 and 2 (for example, a graph showing how much they changed over the four weeks in positive emotions generated from daily life activities).
Sessions 5 and 6 are focused on social interactions in daily life. Again, at the end, they also receive information on their progress in daily life activities (sessions 1 & 2) and daily life events (sessions 3 & 4) accompanied by visual aids: graphs that show changes over the entire six week intervention period.

There are several reasons why we chose to make three different modules:
(1). Information on all items at once is too much, with the ensuing risk that participants get confused and do little or nothing with the information. Therefore, information is built up gradually over the course of treatment, while participants get more and more used to the procedure.
(2). Individuals may habituate quickly (i.e. feel bored) if feedback is received on the same issue over and over again. Now, there are new elements and training possibilities added each two weeks, ensuring “novelty”.
(3). A danger of repeating the feedback on the same items for too long is that the participants get too much insight in the construction of the feedback and will, in the end, fill in socially desired information in their daily life recordings to get positive responses from the researcher. Therefore, feedback is construed differently each two weeks. Since participants will not be informed on future items of feedback they cannot provide socially desired responses. (Another characteristic of the design preventing socially desired responses is that the various feedback results are retrieved through statistical associations between several items which cannot be manipulated readily by participants). Therefore, the study will not formally assess socially desired responding tendencies.

The one constant element of feedback over all six sessions, however, is feedback on the association between patients' average positive emotions as assessed with MET-D and their amount of depressive complaints as assessed with SCL-90 depression scale items (filled in each evening in the MET-D device).

In both treatment arms 1 and 2, participants have weekly sessions with the researcher. In treatment arm 2, however, no feedback on the daily recordings is given. In order to prevent any effects of different duration of researcher contacts in both arms, the difference in time is filled by doing two more interviews on anxiety and depression in treatment arm 2 each week. The structure of the sessions is built up according to a standardized protocol. Clear instructions are formulated on how to give feedback and perform the session in a similar manner for each participant. Training is provided to researchers.
In both arms 1 and 2 there is also a debriefing concerning the MET-D measurements at each session. Discussed are things as: why the participant missed some measurements, whether there are any technical problems, whether some things are unclear etc. Furthermore, the participant is encouraged to fill in the MET-D questions next time as accurately as possible conform the instructions of the researcher.

Levels of reward experience, stress sensitivity and affect variability are calculated from the MET-D observations for each subject at the end of each intervention week. Since these are complex phenotypes derived from combinations of data they are not subject to easy conscious manipulation by patients.

Before entering the study, subjects will be screened for a current depressive episode by means of the Structured Clinical Interview for DSM-IV Axis I Disorders (SCID-I) and the Hamilton Depression Rating Scale (HDRS). After inclusion, a saliva sample will be taken for genetic analysis and the subject has to complete the following measurements: Inventory of Depressive Symptoms (IDS) [31], the NEO personality inventory (NEO-PI-R) [32, 33], Childhood Trauma Questionnaire (CTQ) [34], Interview for Recent Life Events (IRLE) [35], Empowerment Questionnaire [36], TiC-P (Trimbos/iMTA questionnaire for Costs associated with Psychiatric Illness) [37], PRODISQ (PROductivity and DISease Questionnaire) [38], and EQ-5D (Euroqol-5D) [39]. Because we hypothesize that the effect of the MET-D feedback on reward experience results in an increase in of empowerment of the subjects, the Empowerment Questionnaire will be used as an additional measurement of the effect of the MET-D intervention. The latter three questionnaires (TiC-P, PRODISQ and EQ-5D) will be used in the costs-efficacy analysis of the MET-D.

All subjects receive additional MET-D training on a separate moment. All groups receive the following pre and post intervention measurements: i) SCID-I interview for diagnosis of depression, ii) HDRS, and iii) IDS. In addition, there are four follow-up measurements of SCID, HDRS and IDS for all groups at 4, 8, 12 and 24 weeks after the end of the intervention, and two follow up measurements of TiC-P, PRODISQ, and EQ-5D for all groups at 12 and 24 weeks after the intervention (see table 1).

Table 1: Overview of design of the randomized controlled trial

Subjects	Screening	MET-D Instruction	Pre 5 days	6 weeks MET-D intervention 3d/wk	Post 5 days	Follow Up 4, 8, 12, 24 wks
n= 40		*	MET-D	continuous MET-D with feedback + TAU	MET-D	⟶
n= 40		*	MET-D	continuous MET-D without feedback +TAU	MET-D	⟶
n= 40		*	MET-D	TAU	MET-D	⟶

Time frame

t1	t2	t3	t4	t5	t6	t7	t8	t9	t10	t11	t12	t13	t14
•		•						•	•	•	•	•	•
♦													
P								P					
□													□
∇												∇	∇

t number of individual visits for the patient

• SCID-I (after screening: only SCID-I depression), Hamilton Depression Rating Scale (HDRS), Inventory of Depressive Symptoms (IDS), and medication monitor

♦ NEO-PI-R, Childhood Trauma Questionnaire (CTQ), Saliva Sample

P Empowerment Questionnaire

□ Interview for Recent Life Events (IRLE)

∇ TiC-P, PRODISQ, EQ-5D

4. STUDY POPULATION

4.1 Population (base)

A group of depressive patients (n=140) will be recruited within (i) SEARCH, the research network of mental health institutions within Limburg and South-Brabant, The Netherlands, (ii) general practices and (iii) the general population. The patients will be randomly divided into three groups.

4.2 Inclusion criteria

Inclusion criteria are (i) 18-65 years, (ii) a DSM-IV diagnosis of current major depressive disorder or (iii) a past diagnosis of major depression and residual symptoms (HAM-17>7), (iii) current use of antidepressants (all types of antidepressants, thus SSRIs as well as TCAs and MAO inhibitors), (iv) adequate vision, and (v) sufficient Dutch language skills.

4.3 Exclusion criteria

Exclusion criteria are (i) diagnosis of non-affective psychosis (e.g. schizophrenia), schizoaffective disorder, or a bipolar disorder, and (ii) current psychotherapeutic treatment.

4.4 Sample size calculation

Three groups of 40 patients have been chosen since power calculations showed that these sample sizes using repeated measurements yield around 90% power to examine the main hypotheses with power estimates around 88 and 90%. To detect a 3-point difference in HDRS-17 score between the experimental and non-experimental arms with alpha <0.05 and beta<0.10 we have a power of 0.93. To detect an effect of reward experience or stress sensitivity (OR=0.5) on future relapse with alpha <0.05 and beta <0.10 we have a power of 0.95. Since we expect a drop-out of around 20 subjects, we will recruit 140 subjects, leaving 120 participants with complete data randomized over three conditions.

5. TREATMENT OF SUBJECTS

< This chapter is only applicable for intervention studies>

5.1 Investigational product/treatment

The MET-D device is an interactive palmtop that signals subjects at random moments of the day to answer MET-D produced questions about affect and daily events. This new device has recently been patented by our research group and is better equipped than the usual ESM paper-pencil method for the purpose of monitoring since i) it can provide immediate feedback to subjects (it stores all data immediately, can be programmed to perform calculations and to return values to the screen), and ii) it demands less time from subjects to respond to the questions and is therefore more suitable for long-term monitoring.

5.2 Use of co-intervention (if applicable)

Not applicable.

5.3 Escape medication (if applicable)

Not applicable.

6. INVESTIGATIONAL MEDICINAL PRODUCT

<*This chapter is only applicable for research with a medicinal product*>

Not applicable.

7. METHODS

7.1 Study parameters/endpoints

7.1.1 Main study parameter/endpoint

The main objective is to examine if feedback on relevant daily life person-context interactions (reward experience and affect variability as measured with MET-D) during pharmacotherapy results in an immediate and follow-up decrease of depressive symptomatology and relapse and whether this leads to a reduction in health care consumption and costs. Main outcomes therefore are pre – post intervention change in i) depressive symptomatology, ii) ESM phenotypes (reward experience and stress sensitivity), iii) pre – post intervention change on the empowerment questionnaire, and iv) health care consumption and costs. This economic evaluation will involve a combination of a cost-effectiveness analysis (CEA) and a cost-utility analysis (CUA). In a CEA effects are presented in clinical outcomes (in our study decrease depression and relapse). The primary outcomes measure for the cost-utility analysis will be Quality Adjusted Life Years (QALYs), based on the EuroQol utility scores.
Furthermore, to examine whether the effect of the MET-D intervention with feedback leads to a long-term change in above described parameters depressive symptomatology, ESM phenotypes and health care consumption and costs are compared between the three groups on base-line and at 12 and 24 weeks follow up.

7.1.2 Secondary study parameters/endpoints (if applicable)

Furthermore, we want to examine if measurements of dynamic daily life person-context interactions (stress sensitivity, reward experience and affect variability as measured with MET-D) significantly contribute to the prediction of future courses of depressive symptomatology within the time frame of the follow up period. To do so we will compare both baseline scores and difference scores (post-intervention minus baseline) of stress sensitivity, reward experience and affect variability with follow up scores at 4, 8, 12 and 24 weeks for all subjects.

Finally, we want to examine if individual differences in both the effect of feedback on daily life person-context interaction and the predictive value of these person-context interactions can be traced back to genetic differences.

Because the science of psychiatric genetics has become an international effort of worldwide collaboration, mandated by international funding bodies and supported by novel analytic techniques, this protocol has been compiled to assist non-specialist readers.

GWAS approach

Previous experience has learned that the use of candidate gene approaches (i.e. having a specific hypothesis for a specific piece of genetic variation) is a good strategy for disorders with a known pathophysiology, but is insufficient to identify genetic variation for complex disease phenotypes. With a few exceptions, such as Huntington's disease or Parkinson's disease, most phenotypes relevant for the field of neuropsychiatry are considered complex, heterogeneous and of multifactorial causality. Furthermore, their genetic origin is most likely the result of a combination of a large number (>200) genetic variants, each with small effect sizes (Odds ratios around 1.1).

With the advent of new technology that allows to determine sequence variation in about one million genetic markers simultaneously (of the in total approximately 3 million genetic markers that vary between persons), genome-wide association studies (GWAS) have become a new, hypothesis-free research strategy that is especially suited to determine the genetics of complex phenotypes. The use of GWAS in other fields of medicine has resulted in the identification of multiple genetic markers associated with complex phenotypes. For example, more than ten polymorphisms have now been associated with diabetes mellitus type 2, most of which were unknown from candidate gene approaches. GWAS in addition allows for the identification of copy number variants (CNVs), for example deletions, insertions, duplications and complex multi-site variants, that have been shown to be involved in the aetiology of psychiatric disorder.

The drawback of GWAS approaches is that they require huge samples, usually in the order of 20.000 to 60.000 or more subjects, given the degree of multiple testing that needs to be corrected for and the anticipated small effect sizes. Therefore, multi-site international collaboration is the only means to acquire these sample size requirements and MHeNS ascribes to the scientific policy of worldwide sharing of data in order to elucidate the causes of complex disorders. This policy is also mandatory for recipients of funding from the EU, Wellcome Trust, NIH, NIMH, etc. GWAS data collection in

MHeNS is therefore shared anonymously with the various international scientific consortia that study the genetics of schizophrenia, bipolar disorder and other phenotypes and workers of MHeNS are usually involved in the analyses of these data.

Hypothesis-based approach

Since the GWAS research strategy is able to identify small genetic effects on complex phenotypes, but is not able to demonstrate how newly indentified *specific* genetic markers contribute to the disease phenotype, a complementary approach is also needed. This complementary approach is hypothesis-driven involving specific genetic variation, aiming to unravel the genetic contribution of novel identified variants to the disease phenotype. Since insights concerning relevant polymorphisms and haplotypes change rapidly, an up-to-date relational (i.e. cross-relating genotype, phenotypic expressions, biochemical pathways) database relevant for the neurosciences is maintained within MHeNS. The mainly replicatory genetic analyses thus are always matched against the most recent information available worldwide. The scientific approach of GWAS identification followed by hypothesis-based replication also represents a tightly organised international effort: one group identifies a novel variant, followed by immediate efforts to replicate by international groups.

DNA analysis

Therefore, each person's complete set of DNA, or genome, is purified from the blood or cells, placed on tiny chips and scanned on automated laboratory machines. The machines quickly survey each participant's genome for strategically selected markers of genetic variation (around 1 million), which are called single nucleotide polymorphisms, or SNPs. Since information relating to 1 million SNPs is difficult to relate conclusively with any complex phenotype by a single research group, anonymized data will be shared within the framework of international consortia. Secondly, since insights concerning relevant markers change rapidly, the DNA of these individuals is stored for 15 years for determination of previously undetermined genetic markers when there is new information that these markers may be relevant to the phenotype studied.

7.1.3 Other study parameters (if applicable)

Possible moderators of main study parameter:

- number of previous depressive episodes

- previous stress exposure, measured with the Childhood Trauma Questionnaire and the Interview for Recent Life Events
- personality, measured with the NEO personality inventory

Possible confounders/mediators:

- comorbidity (e.g. substance dependence, personality disorders)
- empowerment, measured with the Empowerment Questionnaire
- medication (type, duration of use)

7.2 Randomisation, blinding and treatment allocation

Patients are randomly divided into the ‘MET-D intervention *with* feedback’ group, the ‘MET-D intervention *without* feedback’ group and the ‘control’ group.
Randomization takes place after screening procedures.
Subjects are obviously not blind to their treatment allocation, however, they do remain blind as to how ESM measures relate to testing the hypothesis. Since MET-D feedback will be given to the therapist as well, the therapist is also not blind to the treatment allocation. In order to check that the HDRS ratings by the researcher are not biased by the researcher’s knowledge of the participant’s condition, we compare IDS scores with HDRS ratings. The IDS is a self-report measure with similar item content compared to the HDRS and is proven highly related to clinician’s rated HDRS score[32].

7.3 Study procedures

Subjects are screened for the inclusion criteria, by means of the SCID-I and answer a questionnaire concerning demographic variables. After inclusion subjects:
- undergo a psychiatric interview: HDRS
- fill out additional questionnaires concerning personality (NEO-PI), depression symptomatology (IDS), trauma (Childhood Trauma Questionnaire and Recent Life Event Scale), empowerment (Empowerment Questionnaire), illness and costs due to loss productivity (PRODISQ), illness and illness related costs (TiC-P), and quality of life (Euroquol-5D).
- spit some saliva in special tubes as to measure polymorphisms/haplotypes in the DNA that have been associated in previous studies with the brain reward system and depression.

At base-line:
All subjects will participate in a 5-day MET-D measurement period. After this period, they undergo 2 psychiatric interviews (the depression part of the SCID-I and the HDRS) and

have to complete one self-report questionnaire concerning depressive symptoms (IDS). Subjects are briefed and de-briefed after the ESM period. The ESM debriefing includes the collection of the MET-D devices and discussion concerning unclarities or missing values. See also ‘study design’ for a more elaborate description.

The intervention period:
Subjects randomized to the ‘MET-D intervention *with* feedback’ group will participate in a 6-week-period of 3 days a week of ESM (collected by the MET-D) and receive weekly feedback on these measurements. Subjects randomized to the ‘MET-D intervention *without* feedback’ group will also participate in a 6-week-period of 3 days a week of ESM (collected by the MET-D), but no feedback on the daily recordings is given. In order to prevent any effects of different duration of researcher contacts in both groups, the difference in time is filled by doing two more weekly interviews on anxiety and depression in the ‘MET-D intervention *without* feedback’ group.
All subjects receive TAU as well during the intervention period.

After the intervention period:
All subjects will participate in a second 5-day ESM period, and are again briefed and debriefed with respect to this procedure. Before and after the 5-day ESM period, all subjects again undergo the psychiatric interviews (the depression part of the SCID-I and HDRS) and have to complete the self-report questionnaire concerning depressive symptoms (IDS). The Empowerment Questionnaire has to be completed by all subjects before the 5-day ESM period as well.

Follow up period:
For all subjects, follow-up measurements of SCID-I and HDRS take place at 4, 8, 12 and 24 weeks after the end of the intervention. The follow-up of the Recent Life Event Scale will take place at 12 weeks after the intervention. The follow up of TiC-P, PRODISQ, and EQ-5D will take place at 12 and 24 weeks after the intervention.

7.4 Withdrawal of individual subjects

Subjects can leave the study at any time for any reason if they wish to do so without any consequences. After consulting the independent physician, the investigator can decide to withdraw a subject from the study for urgent medical reasons. Subjects refusing collection of a saliva sample can still participate in the study if the wish to do so.

7.4.1 Specific criteria for withdrawal (if applicable)

Not applicable

7.5 Replacement of individual subjects after withdrawal

In case subjects withdraw before randomization, they will be replaced. After randomization they will not be replaced. By recruiting 20 participants more than the sample size chosen based on power calculations, we take a certain amount of drop-out into account.

7.6 Follow-up of subjects withdrawn from treatment

The number of withdrawals, and reasons for withdrawal will be reported in the publication of the study. It will be examined whether these subjects differ in any characteristics from those that do not withdraw.

7.7 Premature termination of the study

Not applicable.

8. SAFETY REPORTING

Not applicable

9. STATISTICAL ANALYSIS

9.1 Descriptive statistics

The categorical data (e.g. gender, education, employment status, martital status, type of TAU) will be analysed by frequency tables and chi-square tests. Means and standard deviations are used for continuous variables (e.g. age, number of previous depressive episodes).

9.2 Univariate analysis

ESM data (as measured with MET-D) have a hierarchical structure. This means that, multiple observations (beep level) are clustered within subjects (subject level). Multilevel regression analysis is suitable for this type of data because it takes the variability associated with each level of nesting into account [40]. The XTMIXED command in STATA 10.1 (Stata Corporation, College Station, Tex, USA) will be used to perform multilevel linear regression analyses. Analyses will be conducted conform previous work [3, 41-46] using multilevel random regression analyses. Technical details have been described in previous studies [3, 41-46]. As described in previous work [3, 41-46], the following positive affect/negative affect phenotypes of daily life emotional response patterns will be constructed from data collected with the Experience Sampling Method:

i) positive affect (PA)
ii) negative affect (NA)
iii) instability of NA indexed by NA variability in daily life
iv) instability of PA indexed by PA variability in daily life
v) NA response to negatively appraised daily life events (stress-sensitivity)
vi) PA response to positively appraised daily life events (reward experience)

First, to analyse the effect of feedback on immediate and follow-up depression scores and relapse we will compare relevant pre, post and follow-up variables between the three groups using multilevel regression analyses.

In addition, multilevel regression analyses are used to examine whether measurements of dynamic daily life person-context interactions (the above mentioned PA/NA affect phenotypes as measured with MET-D) significantly contribute to the prediction of future courses of depressive symptomatology.

Third, to examine whether individual differences in both the effect of feedback on daily life person-context interaction and the predictive value of these person-context interactions can be traced back to genetic differences, we will analyse the effect of genetic polymorphisms on pre, post and follow-up HDRS, SCID-I by means of multilevel regression analysis.

For the economic evaluation, the Incremental cost-effectiveness ratio (ICER) will be determined on the basis of incremental costs and effects of MET-D (with and without feedback) compared Usual Care. The cost-effectiveness ratio will be stated in terms of costs per outcome rate, the cost-utility ratio will focus on the net cost per QALY gained. The ICER will be calculated as follows. ICER = (Ci – Cc) / (Ei – Ec), where Ci is the annual total cost of the MET-D (with and without feedback) group, Cc is the annual total cost of the Usual Care group, Ei is the effects at one year follow-up for the MET-D (with and without feedback) group and Ec is the effect at one year follow-up for the Usual Care group. The robustness of the ICER will be checked by non-parametric bootstrapping (1000 times) [47]. Bootstrap simulations will also be conducted in order to quantify the uncertainty around the ICER, yielding information about the joint distribution of cost and effect differences. The bootstrapped cost-effectiveness ratios will be subsequently plotted in a cost-effectiveness plane, in which the vertical line reflects the difference in costs and the horizontal line reflects the difference in effectiveness. The choice of treatment depends on the maximum amount of money that society is prepared to pay for a gain in effectiveness, which is called the ceiling ratio. Therefore, the bootstrapped ICERs will also be depicted in a cost-effectiveness acceptability curve showing the probability that MET-D (with and without feedback) is cost-effective using a range of ceiling ratios. Additionally, to demonstrate the robustness of our base-case findings a multi-way sensitivity analyses will be performed. In the sensitivity analysis uncertain factors of assumptions in the base case analysis will recalculated in order to assess whether the assumptions have influenced the incremental cost-effectiveness ratio (ICER), for example by varying cost-prices and volumes between minimum and maximum [47].

9.3 Multivariate analysis

Not applicable.

9.4 Interim analysis (if applicable)

Not applicable.

10 ETHICAL CONSIDERATIONS

10.1 Regulation statement

The study will be conducted according to the principles of the Declaration of Helsinki (2008*)* and in accordance with the Medical Research Involving Human Subjects Act (WMO*).*

10.2 Recruitment and consent

Recruited will take place within (i) SEARCH, the network of mental health institutions within Limburg and South Brabant, (ii) general practices and (iii) the general population. Subjects will be made aware of the possibility to participate in this study by their supervising care givers. Subjects from the general practices and general population will be recruited through posters in drugstores and general practices. Subject will either contact the researcher themselves in case they want to participate or their supervising care giver will contact the researcher. In case subjects show interest they will be invited by the investigator of this study who will provide them with all information about the study and risks, benefits and rights of participants. Subjects have a week to decide whether or not they want to participate. In case a subject does not want to participate, but changes his/her mind later, he or she can still participate.

10.3 Objection by minors or incapacitated subjects (if applicable)

Not applicable. Only adults (>18 years) and capacitated individuals will be included in the study.

10.4 Benefits and risks assessment, group relatedness

There are no health risks associated with the research.

A personal benefit is that the subject may gain increased insight and control over his/her mechanisms of vulnerability and recovery.

A second personal benefit for the subject is that the standard treatment can be adapted to the individual needs since the therapist can use the feedback to assess the patients' development and determine further treatment goals.

There is a third benefit in that the information on (change in) daily life person-context interactions available for the patient and therapist during antidepressant treatment is hypothesized to positively influence treatment efficacy in both the short- and long-term. Furthermore, the continuous monitoring during treatment of these person-context interactive phenotypes, which have been shown to be predictive for risk and recovery of

depression[4], may improve the assessment of treatment response and help clinicians in the estimation of further treatment goals for the particular patient.
When the intervention results in an improvement in treatment efficacy and can be implemented in general mental health care, the benefits for health and quality of life are expected to far outweigh the costs of this study. The cost-effectiveness of the intervention will be explicitly examined. The benefits for society imply a considerable reduction in both the costs of healthcare consumption, the burden on the health care system, and the costs of loss of productivity due to the illness.
Subjects from both control conditions will not be offered the experimental intervention after finishing the study, given the experimental phase of the study.

Burden for the patients is investment of time. After a screening procedure in which subjects have to under go a psychiatric interview, fill out some questionnaires, and also have to spit some saliva in a tube (total of 3 hours), time investment for subjects is 5.5 hours for questionnaires (distributed over 7 meetings). In addition, all subjects participate twice in the MET-D measurements (5-day period of filling in dairies concerning daily life events and mood), which will take approximately 4 hours in total. MET-D measurement takes places in the actual living environment of the patient during which they engage in their normal daily activities, and as such reduces the burden for the patient. Before the start of the MET-D measurements, subjects have to come in for a short meeting of approximately 40 minutes to receive specific instructions related to this procedure and the MET-D device. Subjects in both experimental groups have to complete the MET-D measurements for 3 days a week during a 6-week period, and have weekly contact with the researchers during this 6-weeks period.
In sum, the investment of time after screening procedures and MET-D instruction comprises for all subjects 10 hours. For both experimental groups (MET-D intervention with and without feedback), participation will take an additional 13.5 hours.

10.5 Compensation for injury

The sponsor/investigator has a liability insurance which is in accordance with article 7, subsection 6 of the WMO.

The sponsor (also) has an insurance which is in accordance with the legal requirements in the Netherlands (Article 7 WMO and the Measure regarding Compulsory Insurance for Clinical Research in Humans of 23rd June 2003). This insurance provides cover for damage to research subjects through injury or death caused by the study.

1. € 450.000,-- (i.e. four hundred and fifty thousand Euro) for death or injury for each subject who participates in the Research;
2. € 3.500.000,-- (i.e. three million five hundred thousand Euro) for death or injury for all subjects who participate in the Research;
3. € 5.000.000,-- (i.e. five million Euro) for the total damage incurred by the organisation for all damage disclosed by scientific research for the Sponsor as 'verrichter' in the meaning of said Act in each year of insurance coverage.

The insurance applies to the damage that becomes apparent during the study or within 4 years after the end of the study.

10.6 Incentives (if applicable)

Subjects are fully compensated for any travel expenses related to the study.

11. ADMINISTRATIVE ASPECTS AND PUBLICATION

11.1 Handling and storage of data and documents

Data are handled confidentially and if possible anonymously. The data are coded using a number indicating the order of entry. Only the main investigator (I.M.A. Kramer) has permission to access the source data. Where it is necessary to be able to trace data to an individual subject, a subject identification code list is used to link the data to the subject. The key to the code is safeguarded by the investigator. Collected material is coded and will be stored with the purpose of analysis. After material is analysed it will be destroyed. The handling of personal data complies with the Dutch Personal Data Protection Act (in Dutch: De Wet Bescherming Persoonsgegevens, Wbp).

11.1 Amendments

<*The following text is applicable for studies without an investigational medicinal product*.>
Amendments are changes made to the research after a favourable opinion by the accredited METC has been given. All amendments will be notified to the METC that gave a favourable opinion.

All substantial amendments will be notified to the METC and to the competent authority.

Non-substantial amendments will not be notified to the accredited METC and the competent authority, but will be recorded and filed by the sponsor.

11.3 Annual progress report

The sponsor/investigator will submit a summary of the progress of the trial to the accredited METC once a year. Information will be provided on the date of inclusion of the first subject, numbers of subjects included and numbers of subjects that have completed the trial, serious adverse events/ serious adverse reactions, other problems, and amendments.

11.4 End of study report

<*The following text is applicable for studies without an investigational medicinal product*.>
The investigator will notify the accredited METC of the end of the study within a period of 8 weeks. The end of the study is defined as the last patient's last visit.

In case the study is ended prematurely, the investigator will notify the accredited METC, including the reasons for the premature termination.

Within one year after the end of the study, the investigator/sponsor will submit a final study report with the results of the study, including any publications/abstracts of the study, to the accredited METC.

11.5 Public disclosure and publication policy

Results of this study will be published in peer-reviewed international scientific journals. Both positive and negative results will be published. There are no restrictions in the public disclosure of scientific findings resulting from this study

12. REFERENCES

1. Bijl, R.V., G. van Zessen, and A. Ravelli, Psychiatrische morbiditeit onder volwassenen in Nederland: het NEMESIS-onderzoek. II. Prevalentie van psychiatrische stoornissen. *Nederlands Tijdschrift voor Geneeskunde*, 1997. **141**(50): p. 2453-60.
2. Spijker, J., et al., Duration of major depressive episodes in the general population: Results from the Netherlands Mental Health Survey and Incidence Study (NEMESIS). *British Journal of Psychiatry*, 2002. **181**(3): p. 208-213.
3. Wichers, M., et al., Genetic risk of depression and stress-induced negative affect in daily life. *British Journal of Psychiatry* 2007. **191**: p. 218-23.
4. Wichers, M.C., et al., Reduced stress-sensitivity or increased reward experience: The psychological mechanism of response to antidepressant medication. *Neuropsychopharmacology*, 2008.
5. Wichers, M., et al., Measurements of daily life person-contex interactions prospectively predict future clinical course of depression. submitted.
6. Watson, D. and A. Tellegen, Toward a consensual structure of mood. *Psychological Bulletin*, 1985. **98**(2): p. 219-35.
7. Kessler, R.C., The effects of stressful life events on depression. *Annual Review of Psychology*, 1997: p. 191-214.
8. Kanner, A.D., et al., Comparison of two modes of stress measurement: Daily hassles and uplifts versus major life events. *Journal of Behavioral Medicine*, 1981. ***4***: p. 1-39.
9. Gottesman, I.I. and T.D. Gould, The endophenotype concept in psychiatry: etymology and strategic intentions. *American Journal of Psychiatry,* 2003. **160**(4): p. 636-45.
10. Barge-Schaapveld, D.Q., *Impact of depression and antidepressant therapy in daily life: An experience sampling approach*. 2001, Maastricht: Maastricht University.
11. Csikszentmihalyi, M. and R. Larson, Validity and reliability of the Experience-Sampling Method. *Journal of Nervous and Mental Disease,* 1987. **175**(9): p. 526-36.
12. deVries, M.W., *The experience of psychopathology: Investigating mental disorders in their natural settings*. 1992, Cambridge: Cambridge University Press.
13. Delespaul, P., *Assessing schizophrenia in daily life: The experience sampling method*. 1995, Maastricht: University of Limburg.
14. Peeters, F., et al., Effects of daily events on mood states in major depressive disorder. *Journal of abnormal psychology*, 2003. **112**(2): p. 203-11.
15. Barge-Schaapveld, D.Q. and N.A. Nicolson, Effects of antidepressant treatment on the quality of daily life: an experience sampling study. *Journal of Clinical Psychiatry,* 2002. **63**(6): p. 477-85.
16. Myin-Germeys, I., et al., Emotional reactivity to daily life stress in psychosis and affective disorder: an experience sampling study. *Acta Psychiatrica Scandinavica*, 2003. **107**(2): p. 124-31.
17. Smits, J.A., et al., The efficacy of videotape feedback for enhancing the effects of exposure-based treatment for social anxiety disorder: a controlled investigation. *Behaviour Research and Therapy*, 2006. **44**(12): p. 1773-85.
18. Fichten, C. and J. Wright, Videotape and verbal feedback in behavioral couple therapy: a review. *Journal of Clinical Psychology*, 1983. **39**(2): p. 216-21.
19. Arnkoff, D.B. and J. Stewart, The effectiveness of modeling and videotape feedback on personal problem solving. *Behaviour Research and Therapy*, 1975. **13**(2-3): p. 127-33.
20. Schmidt, U., et al., Does personalized feedback improve the outcome of cognitive-behavioural guided self-care in bulimia nervosa? A preliminary randomized controlled trial. *British Journal of Clinical Psychology*, 2006. **45**(1): p. 111-121.
21. Sullivan, P.F., M.C. Neale, and K.S. Kendler, Genetic epidemiology of major depression: review and meta-analysis. *American journal of psychiatry,* 2000. **157**(10): p. 1552-62.
22. Kendler, K.S., L.M. Thornton, and C.O. Gardner, Genetic risk, number of previous depressive episodes, and stressful life events in predicting onset of major depression. *American Journal of Psychiatry,* 2001. **158**(4): p. 582-6.
23. Kendler, K.S., et al., Stressful life events, genetic liability, and onset of an episode of major depression in women. *American Journal of Psychiatry,* 1995. **152**(6): p. 833-42.

24. Wichers, M., et al., Mechanisms of gene-environment interactions in depression: evidence that genes potentiate multiple sources of adversity. *Psychological Medicine*, 2008. **in press**.
25. Frodl, T., H.J. Moller, and E. Meisenzahl, Neuroimaging genetics: new perspectives in research on major depression? *Acta psychiatrica Scandinavica*, 2008. **118**(5): p. 363-72.
26. Jacobs, N., et al., Genes making one feel blue in the flow of daily life: a momentary assessment study of gene-stress interaction. *Psychosomatic Medicine*, 2006. **68**(2): p. 201-6.
27. Jacobs, N., et al., Stress-related negative affectivity and genetically altered serotonin transporter function: evidence of synergism in shaping risk of depression. *Archives of General Psychiatry*, 2006. **63**(9): p. 989-96.
28. Wichers, M., et al., The psychology of psychiatric genetics: Evidence that positive emotions in females moderate genetic sensitivity to social stress associated with the BDNF Val^{66}Met polymorphism. *Journal of Abnormal Psychology*, 2008. **117**(3): p. 699-704.
29. Smits, K.M., et al., The influence of 5-HTTLPR and STin2 polymorphisms in the serotonin transporter gene on treatment effect of selective serotonin reuptake inhibitors in depressive patients. *Psychiatric genetics*, 2008. **18**(4): p. 184-90.
30. Granholm, E., C. Loh, and J. Swendsen, Feasibility and validity of computerized ecological momentary assessment in schizophrenia. *Schizophrenia Bulletin*, 2008. **34**(3): p. 507-14.
31. Rush, A.J., et al., The Inventory of Depressive Symptomatology (IDS): Psychometric properties. *Psychological Medicine*, 1996. **26**(3): p. 477-486.
32. Young, M.S. and J.A. Schinka, Research Validity Scales for the NEO-PI-R: Additional Evidence for Reliability and Validity. *Journal of Personality Assessment*, 2001. **76**(3): p. 412-420.
33. Schinka, J.A., B.N. Kinder, and T. Kremer, Research Validity Scales for the NEO--PI--R: Development and Initial Validation. *Journal of Personality Assessment*, 1997. **68**(1): p. 127.
34. Bernstein, D.P., et al., Validity of the Childhood Trauma Questionnaire in an adolescent psychiatric population. *Journal of the American Academy of Child & Adolescent Psychiatry*, 1997. **36**(3): p. 340-348.
35. Paykel, E.S., The interview for recent life events. *Psychological Medicine*, 1997. **27**(2): p. 301-310.
36. Boevink, W., H. Kroon, and F. Giesen, *Constructie en validatie van een vragenlijst*. 2008, Utrecht: Trimbos-instituut.
37. Hakkaart-van Roijen, L., *Trimbos/iMTA questionnaire for costs associated with psychiatric illness(TiC-P)*. 2002, Rotterdam: Institute for Medical Technology Assessment.
38. Koopmanschap, M.A., PRODISQ: a modular questionnaire on productivity and disease for economic evaluation studies. *Expert Review of Pharmacoeconomics and Outcomes Research*, 2005. **5**(1): p. 23-28.
39. Prieto, L., et al., A Rasch model analysis to test the cross-cultural validity of the EuroQoL-5D in the Schizophrenia Outpatient Health Outcomes Study. *Acta Psychiatrica Scandinavica*, 2003. **107**: p. 24-29.
40. Snijders, T. and R. Bosker, *Multilevel analysis: an introduction to basis and advanced multilevel modelling.* 1999, London: SAGE publications Ltd.
41. Jacobs, N., et al., A momentary assessment study of the relationship between affective and adrenocortical stress responses in daily life. *Biological Psychology*, 2007. **74**(1): p. 60-66.
42. McConville, C. and C. Cooper, Personality correlates of variable moods. *Personality and Individual Differences*, 1999. **26**: p. 65-78.
43. Myin-Germeys, I. and J. Van Os, Stress-reactivity in psychosis: evidence for an affective pathway to psychosis. *Clinical Psychology Review*, 2007. **27**(4): p. 409-424.
44. Peeters, F., et al., Diurnal mood variation in major depressive disorder. *Emotion*, 2006. **6**(3): p. 383-391.

45. Wichers, M., et al., The catechol-O-methyl transferase Val158Met polymorphism and experience of reward in the flow of daily life. *Neuropsychopharmacology*, 2008. **33**(13): p. 3030-3036.
46. Wichers, M.C., et al., Evidence that moment-to-moment variation in positive emotions buffer genetic risk for depression: a momentary assessment twin study. *Acta Psychiatrica Scandinavica*, 2007. **115**(6): p. 451-7.
47. Briggs, A.H., D.E. Wonderling, and C.Z. Mooney, Pulling cost-effectiveness analysis up by its bootstraps: a non-parametric approach to confidence interval estimation. *Health Economics*, 1997. **6**(4): p. 327-340.
